# Exome analysis identifies Brody myopathy in a family diagnosed with malignant hyperthermia susceptibility

**DOI:** 10.1002/mgg3.91

**Published:** 2014-06-06

**Authors:** Nyamkhishig Sambuughin, Elena Zvaritch, Natasha Kraeva, Olga Sizova, Erica Sivak, Kelley Dickson, Margaret Weglinski, John Capacchione, Sheila Muldoon, Sheila Riazi, Susan Hamilton, Barbara Brandom, David H MacLennan

**Affiliations:** 1 Defense and Veterans Center for Integrated Pain Management, Henry M. Jackson FoundationRockville, Maryland; 2 Department of Anesthesiology, Uniformed Services UniversityBethesda, Maryland; 3 Banting and Best Department of Medical Research, University of TorontoToronto, Ontario, Canada; 4 Department of Anesthesia, Toronto General HospitalToronto, Ontario, Canada; 5 Department of Anesthesiology, Children's Hospital, University of PittsburghPittsburgh, Pennsylvania; 6 Department of Anesthesiology, Mayo ClinicRochester, Minnesota; 7 Department of Molecular Physiology and Biophysics, Baylor College of MedicineHouston, Texas

**Keywords:** Brody myopathy, malignant hyperthermia, RYR1, SERCA1

## Abstract

Whole exome sequencing (WES) was used to determine the primary cause of muscle disorder in a family diagnosed with a mild, undetermined myopathy and malignant hyperthermia (MH) susceptibility (MHS). WES revealed the compound heterozygous mutations, p.Ile235Asn and p.Glu982Lys, in *ATP2A1*, encoding the sarco(endo)plasmic reticulum Ca^2+^ ATPase type 1 (SERCA1), a calcium pump, expressed in fast-twitch muscles. Recessive mutations in *ATP2A1* are known to cause Brody myopathy, a rare muscle disorder characterized by exercise-induced impairment of muscle relaxation and stiffness. Analyses of affected muscles showed the absence of SERCA1, but SERCA2 upregulation in slow and fast myofibers, suggesting a compensatory mechanism that partially restores the diminished Ca^2+^ transport in Brody myopathy. This compensatory adaptation to the lack of SERCA1 Ca^2+^ pumping activity within the muscle explains, in part, the mild course of disease in our patient. Diagnosis of MHS in this family was secondary to a loss of SERCA1 due to disease-associated mutations. Although there are obvious differences in clinical expression and molecular mechanisms between MH and Brody myopathy, a feature common to both conditions is elevated myoplasmic Ca^2+^ content. Prolonged intracellular Ca^2+^ elevation is likely to have led to MHS diagnosis in vitro and postoperative MH-like symptoms in Brody patient.

## Introduction

Skeletal muscle contraction and relaxation are tightly regulated by the myoplasmic calcium (Ca^2+^) level. Ca^2+^ released from the sarcoplasmic reticulum (SR) through the skeletal muscle Ca^2+^ release channel ryanodine receptor type 1 (RYR1) binds directly to troponin C in thin filaments, forming the “on” signal for induction of muscle contraction (MacLennan 2000). The “off” signal, that induces muscle relaxation, is the pumping of Ca^2+^ from the myoplasm back into the SR by sarco/endoplasmic Ca^2+^ ATPases (SERCA pumps). Ca^2+^ reuptake creates a Ca^2+^ store in the lumen of SR for cyclical Ca^2+^ signaling in the myofibers. Two SERCA isoforms are expressed in mammalian muscles: sarco(endo)plasmic reticulum Ca^2+^ ATPase type 1 (SERCA1) is the major isoform in fast-twitch (type 2) skeletal muscle fibers, whereas SERCA2 is predominantly expressed in cardiac and slow-twitch (type 1) skeletal muscle fibers (MacLennan 2000; Brini and Carafoli 2009).

Mutations in the genes encoding RYR1 (*RYR1*) and SERCA1 (*ATP2A1*) are known to cause abnormal Ca^2+^ regulation: *RYR1* mutations cause malignant hyperthermia (MH) (MIM: 145600); *ATP2A1* mutations cause Brody myopathy (MIM: 601003) (Odermatt et al. 1996; Rosenberg et al. 1997; Maclennan and Zvaritch 2011). These two muscle disorders differ in their clinical presentation, inheritance pattern, and prevalence. MH typically manifests as a drug-induced, severe metabolic reaction in susceptible individuals upon administration of potent inhalation anesthetics and/or depolarizing muscle relaxants. MH susceptibility (MHS) is inherited as an autosomal dominant trait with reports of over 500 different causal *RYR1* mutations (Rosenberg et al. 1997; Maclennan and Zvaritch 2011). Most MHS individuals are asymptomatic and lack clinical symptoms until challenged by anesthetics. Brody myopathy is an autosomal recessive disorder, characterized by muscle stiffness, cramps, with and without pain, and progressive impairment of muscle relaxation during exercise (Brody 1969; Odermatt et al. 1996; Voermans et al. 2012). Brody myopathy is extremely rare; to date, only eight families with causal *ATP2A1* mutations have been reported (Odermatt et al. 1996; Odermatt et al. 2000; Vattemi et al. 2010; Voermans et al. 2012; Guglielmi et al. 2013).

In this article, we describe two siblings with new compound heterozygous mutations in *ATP2A1*. The index case of the family was initially referred for genetic analysis of MH due to postoperative rigidity; subsequent caffeine-halothane contracture tests (CHCT) were positive for MHS. We present clinical, genetic, and biochemical evidence that the siblings were suffering from Brody myopathy. Protein analyses showed near absence of SERCA1 and a significant twofold increase in SERCA2 content in affected muscle that was confirmed by immunofluorescent confocal microscopy showing SERCA2 upregulation in both slow- and fast-twitch fibers, providing the first clear evidence of a compensatory mechanism that partially restores diminished Ca^2+^ transport in Brody myopathy. This compensatory adaptation to the lack of SERCA1 Ca^2+^ pumping activity within the muscle explains the mild course of disease in our patients. MHS diagnosis in this family was secondary to a loss of SERCA1 due to disease-associated mutations. We discuss the relationship between Brody myopathy and MHS, emphasizing challenges in clinical and molecular genetic diagnostics.

## Material and Methods

The study was approved by the Institutional Review Boards of the participating institutes. After obtaining informed consents, the index case and his sister were enrolled in the genetic study.

### Patients and muscle biopsy

A 67-year-old male patient with a history of muscle pain, weakness, heat intolerance, and inability to run had cardiac surgery with no adverse reaction to anesthetics. However, he developed skeletal muscle rigidity after an operation and his creatine kinase level was slightly elevated. The patient's sister complained of muscle weakness and pain, inability to run, and considered herself and her brother to have been “handicapped” since childhood. She was prescribed a muscle relaxant, which improved her symptoms. She did not complain of heat intolerance. She has had three children who are all able to run and participate in sports. She stated that her parents were farmers and did not have any muscle symptoms.

Biopsies were taken from the following muscles: for index case *vastus lateralis* and for control individuals *gracilis*, and stored at −70°C. Prior to freezing, the index case muscle biopsy was kept in Ringer solution during the whole time of CHCT procedure, up to 3 h at room temperature (RT). For consistency, control muscle samples were collected from biopsy specimen kept under similar conditions, unless otherwise specified. Control individuals (*n* = 4) were unrelated healthy adult males that were diagnosed MH negative (MHN) by CHCT.

#### Histopathology and muscle contracture test

The muscle pathology report of the index case showed an increase in the proportion of fibers containing internal nuclei. Necrotic or regenerating fibers were not observed. Inflammatory changes were absent. There was an increase in connective tissue endomysial and perimysial spaces. In muscle sections stained for NADH dehydrogenase activity, a few fibers exhibited decreased enzyme activity near their centers. Scattered cytochrome c oxidase negative fibers were present. In sections stained for ATPase, type 2A fibers were smaller in mean diameter and essentially all of the atrophic fibers were histochemically type 2A. The patient was diagnosed with mild, undetermined myopathy. Because of postoperative rigidity, the patient was referred for MHS testing, which was performed according to the North American CHCT protocol (Larach 1989). The maximum increases from baseline tensions were 5.74 and 1.31 g to 3% halothane and to 2 mmol/L caffeine, respectively, and the patient was diagnosed as MHS. There was no family history of anesthetic complications, and the subject had three uneventful surgeries under general anesthesia prior to his heart surgery. All of his siblings, including the affected sister, had received general anesthesia without complications.

### Genetic analysis

#### Mutation screening of RYR1 and ATP2A1

Genomic DNA (gDNA) was extracted from peripheral blood and saliva using standard protocols. Complete sequencing of *RYR1* was performed in the index case. A cohort of 50 independent subjects diagnosed as MHS by positive contracture test results was screened for the entire ATPase, Ca^2+^-transporting, fast-twitch 1 (*ATP2A1*; MIM: 108730) gene. Primers used for screening of the entire *RYR1* were published previously (Groom et al. 2011). Primers for screening of *ATP2A1* exons were designed using Primer3 software (http://frodo.wi.mit.edu/primer3/) and available upon request. Both genes were screened by Sanger sequencing and results were analyzed by Sequence Scanner Software (ABI, v.1.0) and Basic Local Alignment Search Tool (BLAST, NCBI, NIH, Bethesda, MD). The p.Lys1393Arg mutation in exon 29 of *RYR1* was screened in a cohort of 107 independent subjects diagnosed as MHN. Mutation analysis was performed by digesting amplified exon with restriction enzyme BstNI, followed by validation with Sanger sequencing.

#### Whole exome sequencing

The gDNA sample from the index case was subjected to whole exome sequencing (WES). WES was performed at Edgebio (EgdeBio, Gaithersburg, MD). The analysis framework to select missense single nucleotide polymorphisms and coding insertions/deletions that are not present in publicly available exome databases and to determine variant impact was used as described (Sambuughin et al. 2013; Toro et al. 2013). Mutation impact was determined with PolyPhen, SIFT, and Mutation Accessor. Novel mutations identified by WES were validated with standard Sanger sequencing of amplified DNA fragments in both affected siblings.

#### Segregation analysis of the Ile235Thr and Glu982Lys mutations in APT2A1

A 2386 bp fragment of the human *ATP2A1* transcript encoding SERCA1 amino acids 235–982 was generated using skeletal muscle mRNA from the index case. Reverse transcription was conducted with forward primer: 5′-ACCAGGACAAGAAGAACATGC-3′; reverse primer: 5′-TAGGTAGTTCCGAGCAACGAA-3′, using SuperScript Reverse Transcriptase (Life Technologies, Grand Island, NY), followed by PCR reactions with long fragment DNA polymerase (Takara, Mountain View, CA). PCR products were then subcloned directly into competent cells using the TOPO-TA cloning kit (Life Technologies). Plasmid DNA from 16 colonies was sequenced, 13 contained the sequence encoding amino acids 235–982. Among analyzed colonies, eight contained the sequence encoding the lle235Asn mutation and five contained the sequence encoding the Glu982Lys mutation. No colonies were found to contain plasmid DNA with both mutations and none contained the wild-type sequence only.

#### Screening of RYR1 transcript

The entire *RYR1* transcript was generated using skeletal muscle mRNA extracted from the index case. Reverse transcription reactions to synthesize cDNA were described in the previous section. Overlapping cDNA fragments were amplified and sequenced using Sanger sequencing.

### Protein analysis

SDS-gel electrophoresis and Western blot analysis of protein in whole-muscle homogenates were performed as described in (Kraeva et al. 2013) with modifications. Briefly, frozen muscle samples (∼150 mg) were powdered under liquid nitrogen and homogenized in 0.6-mL ice-cold buffer, containing 50 mmol/L Tris-HCl (pH 7.4); 10 mmol/L EGTA; 2 mmol/L EDTA; 5 mmol/L DTT; 0.5 mmol/L PMSF, and protease inhibitor cocktail (Complete mini, Roche Diagnostics, Indianapolis, IN). Muscle homogenates (0.5–20 μg total protein) were mixed with 2x SDS sample buffer and boiled for 5 min prior to protein separation on 5–15% Mini-PROTEAN TGX™ SDS-PAGE gels (Bio-Rad Laboratories, Mississauga, ON, Canada). BLUeye Prestained Protein Ladder (GeneDireX, Atlanta, GA) was used as a protein molecular weight marker. Following electrophoresis, the proteins were transferred onto Immobilon P^SQ^ membranes (0.2-μm pore size; EMD Millipore, Billerica, MA). The membranes were blocked with 5% (w/v) nonfat dry milk in 10 mmol/L PBS, pH 7.2, for 1 h at RT and incubated with one of the following mouse monoclonal IgG antibodies: anti-panRYR (1:250; 34°C, Thermo Scientific, Rockford, IL) and anti-RYR1 (1:1000, XA7B6; Upstate Cell Signaling Solutions, Lake Placid, NY); anti-SERCA1 (1:24,000, VE121G9; Abcam, Toronto, ON, Canada) and anti-SERCA2 (1:20,000, IID8; Sigma, Oakville, ON, Canada). SERCA1 protein expression was also tested with polyclonal goat anti-human SERCA1 antibody, N-19 (1:200; Santa Cruz Biotechnology, Dallas, TX) and with mouse monoclonal antibody A52 (Zubrzycka-Gaarn et al. 1984).

Following incubation with the corresponding, anti-mouse IgG (1:1000; Sigma-Aldrich, Oakville, ON, Canada) and anti-goat IgG (1:1000; Sigma) antibodies, conjugated to horseradish peroxidase. The immune complexes were revealed using chemiluminescent Luminata™ Forte Western HRP substrate (EMD Millipore). For loading controls, the blots were reprobed with at least one of the following antibodies: mouse monoclonal anti-*α*-tubulin IgG (1/200, 6A204; Santa Cruz Biotechnology), mouse monoclonal anti-*α*-actin IgM (1:60,000, 5C5; Sigma), and rabbit polyclonal anti-GAPDH (1:3000; Millipore). The images were generated using Fluo S™ Max MultiImager and Quantity One software (Bio-Rad Laboratories). Densitometry was performed with the ImageJ software (National Institute of Health, Bethesda, MD).

### Confocal microscopy

Longitudinal and transverse cryostat sections (7 μm) of control and Brody patient skeletal muscle biopsy were fixed in 4% PBS buffered formaldehyde, 5 min at RT. After blocking for 1 h at RT in PBS, containing 10% chicken serum (Life Technologies), 0.1% Triton X100 (Sigma), and 1% BSA (Bio-Shop, Canada Inc), the sections were incubated with mouse monoclonal antibodies against fast- and slow-twitch skeletal muscle myosins (1/200; Sigma) and SERCA2 (1:50, Sigma) overnight at 4°C. For visualization of connective tissue, the sections were costained with wheat germ agglutinin (WGA) Alexa Fluor 633 conjugate (WGA-Alexa, 1 μg/mL; Life Technologies). Sections were then washed (4 × 5 min) in PBS, containing 0.05% Tween 20 (PBS-T; Sigma) and incubated with secondary antibodies – chicken anti-mouse IgG1 Alexa Fluor 488 (1:1000; Life Technologies) for 1 h at RT. Sections were washed (3 × 5 min) in PBS-T and mounted with anti-fade UltraCruz Mounting media containing DAPI (Santa Cruz) and viewed under Olympus Fluoview B a laser scanner microscope. The images were analyzed using Olympus FV100 version 3.1 image viewer software (Olympus, Center Valley, PA).

### Data analysis and statistics

The results are expressed as mean ± SEM obtained from multiple determinations in at least three separate experiments. The significance of changes was evaluated using the unpaired Student's *t*-test. The *P *< 0.05 was considered significant.

## Results

### Genetic analysis and whole exome results

**Figure 1:**
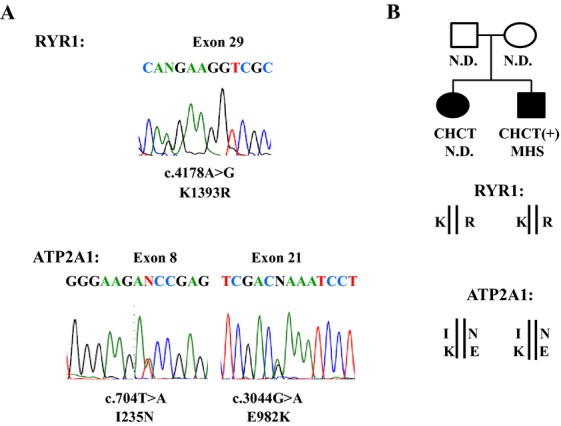
(A) Sequence chromatographs of *RYR1* and *ATP2A1* in the index case. Positions of nucleotide changes (N) those result in p.Lys1393Arg (K1393R) in *RYR1* and p.Ile235Asn (I235N) and p.Glu982Lys (E982K) in *APT2A1*. (B) Segregation of alleles in the affected siblings. Filled symbols indicate affected individuals. CHCT denotes caffeine and halothane contracture test. N.D. indicates not determined. Empty symbols indicate clinically healthy parents.

The MHS diagnosis for the index patient prompted us to search for a mutation in *RYR1*. Genetic analysis of both the index case and his affected sister identified a heterozygous c.4178A>G; p.Lys1393Arg mutation in exon 29 of *RYR1* (NM_001042723) (Fig.1A). The p.Lys1393Arg mutation was reported previously in association with MHS, exertional rhabdomyolysis, and axial myopathy (Broman et al. 2009; Dlamini et al. 2013; Løseth et al. 2013). The p.Lys1393Arg mutation is also reported in the general population at a frequency ≥1% (Broman et al. 2009; Gonsalves et al. 2013; Exome Variant Server at http://evs.gs.washington.edu/EVS/), which is well above the estimated frequency of MHS-associated mutations in *RYR1* (Rosenberg et al. 1997; Brandom et al. 2013). Because the general population cannot truly present the MHN control population, we have screened 107 subjects with MHN diagnosis for the p.Lys1393Arg mutation. The results revealed that three MHN controls carried the mutation, suggesting that the association of the p.Lys1393Arg mutation with MHS is weak. Of most importance in considering the association of this mutation with disease in our family, the highly penetrant childhood onset of muscle disease in two affected siblings, with no history of muscle disease in either parent, cannot be explained by the presence of a familial, dominantly inherited *RYR1* mutation. A deep intronic mutation causing faulty splicing has been found in a RYR1-related myopathy and these types of mutations can be missed by gDNA screening (Monnier et al. 2008). To determine whether any such changes might have been missed during gDNA screening, the *RYR1* transcript was also analyzed, the results of the entire *RYR1* transcript analysis were negative.

To identify the true underlying cause of the myopathy, we turned to WES. WES was performed on gDNA from the index case, which resulted in about 87% of the targets reaching >20× coverage. WES analysis confirmed the presence of the p.Lys1393Arg in *RYR1* and showed negative results for other MH causative gene, *CACNA1S* (Rosenberg et al. 1997; Maclennan and Zvaritch 2011), encoding the *α*1-subunit of the skeletal muscle Ca^2+^ channel and *CASQ1* encoding calsequestrin, a causal factor of an MH-type reaction in mice (Dainese et al. 2009). A new, c. 2974C>T, p.His992Tyr, mutation was detected in *CACNA2D1* (NM_000722). Although this gene encodes the *α*2/∂ subunit of the skeletal muscle Ca^2+^ channel and is located within a chromosomal region linked to MHS (Iles et al. 1994; Robinson et al. 2003), a causal role for this mutation was ruled out on the basis of its predicted dominance and its predicted benign effect, but mainly on the basis of its absence in the affected sibling.

Among additional novel missense mutations identified in the index case, we considered c.2125G>T, p.Gly709Stop mutation in the *KIAA0196* (NM_014846) encoding strumpellin as potentially significant, since this mutation was shared by both affected siblings. It introduced a stop codon mutation that would likely lead to protein truncation. Dominant mutations in *KIAA0196* are associated with an aggressive subtype of spastic paraplegia, SPG8 (Valdmanis et al. 2007), characterized by spastic gait, spasticity of lower limbs, hyperreflexia, and decreased vibration sensation, with an age of onset between 20 and 30 years. None of these symptoms corresponded to the symptoms of our two affected siblings, who exhibited awkwardness and exercise-induced contractures in their earliest childhood. In addition, all known disease-associated *KIAA0196* mutations are missense and no truncating mutations have been reported in SPG8 patients, suggesting that gain of function rather than loss of function mutations is a highly relevant feature of the pathogenesis of this disorder. Therefore, an association of the *KIAA0196* p.Gly709Stop with myopathy in our family was ruled out.

We identified two new heterozygous missense mutations, c.704T>A; p.Ile235Asn in exon 8 and c.2944G>A; p.Glu982Lys in exon 21, in *ATP2A1* (NM_004320) (Fig.1A). These mutations have not been reported in ClinSeq, 1000 genomes databases, or the publicly available database of >6500 European and African American exomes (Exome Variant Server: http://evs.gs.washington.edu/EVS/). The two mutations were confirmed by Sanger sequencing in both the index case and his affected sister. Both mutations were predicted to have damaging effects by three different predictive programs.

To characterize how these two mutations segregate within our family, we cloned *ATP2A1* transcripts isolated from the index case. Sequencing of *ATP2A1* clones showed that p.Ile235Asn and p.Glu982Lys occurred as compound heterozygotes, inherited in a recessive fashion (Fig.1B). The inheritance of recessive mutations in *ATP2A1* has been shown previously to cause Brody myopathy, which presented in childhood and caused exercise-induced impairment of muscle relaxation and stiffness with and without muscle weakness and pain (Brody 1969; Voermans et al. 2012). These are the signs and symptoms that characterize the clinical features of our affected siblings. Because the index case was diagnosed with MHS, we screened the entire *ATP2A1* in 50 independent MHS subjects. No significant *ATP2A1* mutation was identified in any of these patients (Table S1).

#### Mutation localizations in the SERCA1

**Figure 2:**
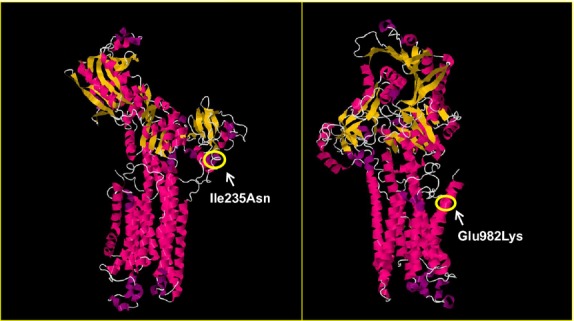
Disease-associated mutations in the Ca^2+^ bound rabbit sarco(endo)plasmic reticulum Ca^2+^ ATPase type 1 crystal structure (1SU4P) from the Protein Data Bank (www.rcsb.org/pdb). The positions of two new mutations identified in this study are circled.

The SERCA1 protein consists of a single polypeptide chain folded into three major domains: actuator (A); nucleotide binding (N); phosphorylation (P); and 10 transmembrane (TM) helices (MacLennan et al. 1998; Brini and Carafoli 2009). Ile 235 and Glu 982 are highly conserved in SERCA1 throughout different species (data not shown). Ile 235 is located in the A domain where the majority of *ATP2A1* mutations associated with Brody myopathy have been found (Fig. S1). Ile 235 lies at the beginning of the *α*3 helix of the A domain (Fig.2), where a change from a nonpolar isoleucine to a polar asparagine may have a destabilizing effect on SERCA1 function. Glu 982 lies within the last TM domain (TM10) of the protein (Fig.2). In this position, a change from a negatively charged glutamate to a positively charged lysine is also likely to have a damaging effect on the stability of SERCA1.

#### Protein analysis

**Figure 3:**
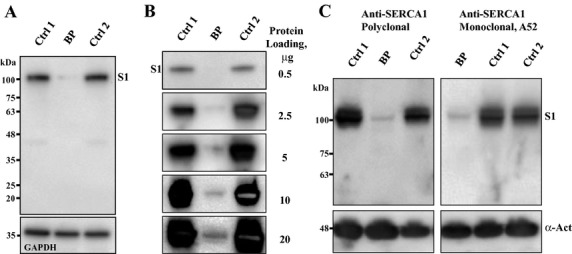
Western blot analysis of sarco(endo)plasmic reticulum Ca^2+^ ATPase type 1 (SERCA1). Ctrl, control samples; BD, patient sample. (A) Almost complete absence of SERCA1 (S1) was revealed in Brody patient compared to control samples. (B) Semiquantitative analysis of SERCA1 protein expression using mouse monoclonal IID8 anti-SERCA1 antibodies. The amounts of total loaded protein in the muscle homogenates are indicated on the right. In the Brody muscle, a well-defined SERCA1 protein band is revealed only at high protein loadings of 10 and 20 μg protein. At similar loadings, control samples show overloaded and oversaturated SERCA1 protein bands. (C) Analysis of muscle homogenates using goat polyclonal (left panel) and mouse monoclonal A52 (right panel) anti-SERCA1 antibodies.

To determine the effect of *ATP2A1* mutations on muscle protein integrity, we analyzed the expression of SERCA1 and related proteins in skeletal muscle biopsies from the index case and healthy adult male subjects (*n* = 4) diagnosed as MHN by MH contracture tests. Western blot analysis of whole-muscle protein extracts with a monoclonal anti-SERCA1 antibody VE121G9 revealed a dramatic decrease in SERCA1 protein content in our Brody patient muscle (Fig.3A). Densitometric analysis of semiquantitative Western blots showed a 20-fold (19.6 ± 3.3, *n* = 6, *P *< 0.05) reduction in SERCA1 protein in the muscle of our Brody patient (Fig.3B). The near absence of SERCA1 was further confirmed by Western blot analysis using two additional SERCA1-specific antibodies: a mouse monoclonal, A52; and goat polyclonal antibody, N-19, directed against the *N*-terminal, human-specific SERCA1 peptide (Fig.3C). Neither of these antibodies revealed any appreciable amounts of the full-length SERCA1 protein or any products of SERCA1 fragmentation in the broad range of the polypeptide mass from 110 to 10 kDa, arguing against SERCA1 degradation following muscle harvesting as a possible cause of its absence.

**Figure 4:**
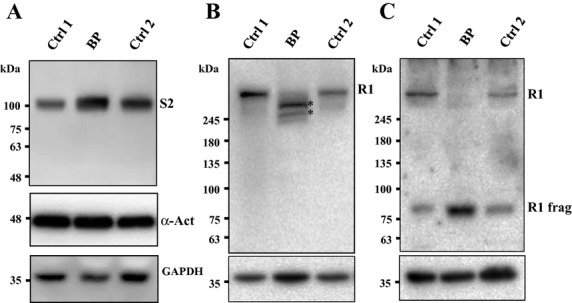
Western blot analysis of sarco(endo)plasmic reticulum Ca^2+^ ATPase type 2 (SERCA2) and ryanodine receptor type 1 (RYR1) expression in skeletal muscle biopsy of the Brody patient (BP) and two healthy control individuals (Ctrl1 and 2). One (A) and 10 mg (B–C) total protein from whole-muscle homogenates were resolved on 4–15% gradient SDS-gels. (A) SERCA2 (S2) protein expression is increased almost twofold in the BP muscle. (B) Anti-RYR 34C antibodies fail to detect the full-length RYR1 (R1) protein in the BP muscle but reveal well-defined polypeptide bands of a lower molecular mass (asterisks) indicating RYR1 proteolysis. (C) Anti-RYR1 XA7 antibodies fail to reveal the full-length RYR1, but detect a lower molecular mass fragment of about 80 kDa (R1 frag). Immunodetected bands of *α*-actin (A) and GAPDH (A–C) were used as loading controls. Molecular mass standards are indicated on the left of each panel.

The SERCA2 level in our Brody patient muscle, compared to control muscle samples, was estimated relative to a set of loading control proteins: *α*-actin, *α*-tubulin, GAPDH, and HPRT, through semiquantitative Western blot analysis (Fig.4A). Relative estimates using different loading controls produced essentially the same results, indicating a significant, almost twofold increase in SERCA2 expression levels (1.8 ± 0.24, *n* = 6, *P *< 0.05), compared to control muscle samples.

Western blot analysis for RYR1 was performed using 34C pan-RYR antibody with an epitope mapped to amino acids 2756–2803 of the human RYR1 sequence (Meng et al. 2007). Surprisingly, the full-length RYR1 band that was readily detectable in control samples was not observed in the Brody patient muscle sample (Fig.4B). Instead, two major RYR1-specific protein bands of slightly lower molecular mass, estimated to be 350 and 420 kDa were detected. In addition, staining with anti-RYR1 antibody XA7 (Paul-Pletzer et al. 2005) directed against amino acids 590–609 in the *N*-terminal portion of the protein showed a single immunoreactive band of about 80 kDa (Fig.4C) that was also present in much lower amounts in control muscle samples.

The finding of RYR1 proteolysis in our patient is inconsistent with the fact that there was no obvious impairment of excitation–contraction coupling in either of the two siblings, which would imply that RYR1 had been fully functional throughout their lives. The combined antibody staining density revealed roughly the same amount of total RYR-specific immunoreactive material in the muscle samples of the index patient and control subjects. This suggests that proteolysis observed in the muscle samples from our Brody patient took place upon muscle harvesting and was limited to as few as two sites near the *N*-terminal end of the RYR1 polypeptide. This proteolysis appeared to be specific for RYR1, since we did not observe any proteolysis of SERCA2 or any of the loading control proteins (Fig.4).

Ryanodine receptor type 1 protein is known to be susceptible to partial proteolysis by Ca^2+^-dependent proteases such as calpain upon skeletal muscle sample excision and handling (Wu et al. 1997; Tompa et al. 2004), but in all cases reported the full-length RYR1 protein has been present as the major immunoreactive band in Western blots. The muscle sample from our index patient was from a muscle biopsy used in CHCT analysis. Prior to freezing, the sample was kept for about 3 h at RT in oxygenated Ringer solution. To test whether complete loss of the full-length RYR1 protein could be reproduced in control MHN muscle samples, incubated for a prolonged period at RT in Ringer solution or exposed to the CHCT test, we performed Western blot analysis of MHN control muscle samples exposed to these conditions, which might have evoked RYR1 proteolysis (Fig. S2). We observed some proteolysis of RYR1, but not the complete disappearance of the full-length RYR1 protein in these control samples. Thus, we concluded that extensive RYR1 proteolysis observed in the Brody patient muscle was specific to RYR1 itself and, moreover, that it was specific to the conditions existing in the patient's muscle. We do not think that it was related to the heterozygous presence of the p.Lys1393Arg mutation, since this should affect only 50% of the RYR1 protein. However, tetramer formation might have unanticipated effects on proteolytic sensitivity. A more attractive alternate possibility is that prolonged elevation of Ca^2+^ in the muscle, resulting from the loss of SERCA activity might have activated a Ca^2+^-dependent protease, such as calpain, that would degrade RYR1 with the high specificity that we observed.

#### Confocal microscopy

**Figure 5:**
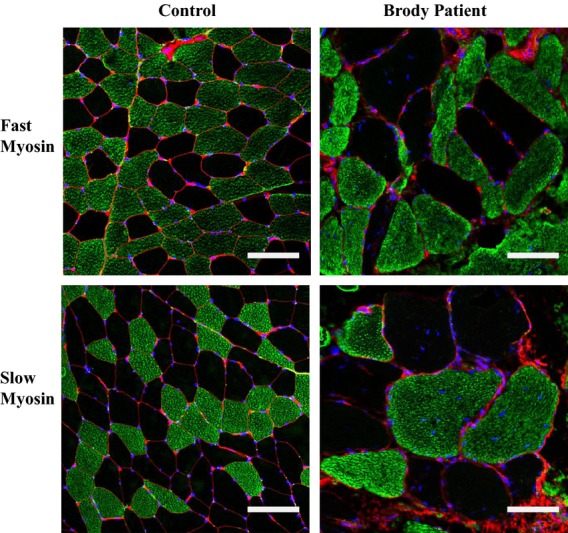
Immunofluorescence staining for fast and slow myosins in the muscle of Brody patient (BP). Confocal microscopy images of transverse skeletal muscle sections stained with anti-fast (top) and anti-slow (bottom) myosin antibodies (green). WGA (red) stains connective tissue. DAPI (blue) counterstains nuclei. Increased fiber size variability and fiber hypertrophy is readily observed in both types of the BP muscle fibers. Bar, 100 μm.

**Figure 6:**
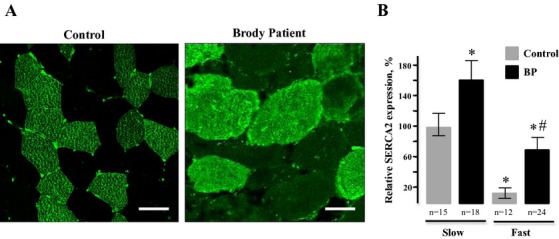
Analysis of sarco(endo)plasmic reticulum Ca^2+^ ATPase type 2 (SERCA2) immunoreactivity in myofibers of Brody patient (BP). (A) Representative immunofluorescence confocal microscopy images of transverse cryostat sections from a control (left) and our BP (right) skeletal muscle biopsy stained with anti-SERCA2 antibodies (green). The sections were processed in parallel and the images were taken at identical microscope and laser intensity settings. The control muscle shows a characteristic pattern of SERCA2 expression that is restricted to slow-type myofibers, while the fast-type myofibers that do not express SERCA2 remain unstained and appear dark. In the muscle section from our BP (right), SERCA2 immunoreactivity is detected in both fast and slow myofibers. Bar, 50 μm. (B) Semiquantitative analysis of SERCA2-specific immunofluorescence reactivity in fast- and slow-type myofibers of the control and BP muscles relative to SERCA2 reactivity in the control slow myofibers. Vertical bars represent standard error of the mean. The number of samples (*n*) is the number of fibers assessed for each group. *Significantly different compared with control slow fibers (*P *< 0.05). ^#^Significantly different compared with control fast fibers (*P* < 0.05).

We addressed whether SERCA2 upregulation occurred in fast fibers that lack SERCA1 or in slow fibers that express SERCA2 normally by immunostaining of affected muscle. Immunofluorescence staining of our Brody patient muscle sections for slow- and fast-twitch myosins confirmed previous histological findings of marked variation in fiber size and increased number of internal nuclei (Fig.5). Both types of fibers exhibited hypertrophy, but it was most prominent in slow fibers, the mean diameter being three- to fourfold greater than those in controls. Fast fibers showed high-fiber size variability and up to 90% of them had multiple, centrally located nuclei that were abnormally elongated. Immunofluorescence staining of our Brody patient muscle sections with anti-SERCA2 antibodies revealed higher than normal immunoreactivity in both fast and slow fibers (Fig.6A). We observed a 1.5- to twofold increase in SERCA2-specific immunoreactivity in Brody slow fibers compared to control slow fibers, whereas the SERCA2-specific immunostaining within the Brody fast fibers showed a significant fourfold increase compared to the control fast fibers (Fig.6B). The elevated SERCA2 expression in fast fibers lacking SERCA1 supports the idea that a compensatory mechanism is activated in these myofibers, representing a novel finding in Brody disease pathology.

## Discussion

We describe a family in which two affected siblings presented with childhood onset exercise intolerance, including the inability to perform repeated tasks such as running, muscle pain, and weakness. The parents and children of the siblings were healthy, suggesting that inheritance of the myopathy was recessive. There was no familial history of any anesthetic complications, but the proband developed postoperative rigidity followed by a positive MH contracture test, which resulted in his diagnosis as MHS. Candidate gene screen, followed by exome analysis, revealed that the proband and his affected sister inherited mutations in two genes that have been associated with two different muscle diseases. A search of *RYR1* that is associated with the majority of dominantly inherited MH cases revealed the presence of a heterozygous p.Lys1393Arg mutation in both affected siblings. We have ruled out the possibility that this mutation is the cause of myopathy in the affected siblings, since the muscle disease observed in our family was inherited recessively.

Whole exome sequencing revealed the presence of two recessively inherited compound heterozygous mutations in *ATP2A1* that would encode for p.Ile235Asn and p.Glu982Lys in SERCA1. Both of these *ATP2A1* mutations are newly identified in this study and are absent in >6500 exomes. They change highly conserved residues in SERCA1, with effects that are predicted by various analyses to be damaging. The locations of both mutations in the protein structure show that p.Ile235Asn is likely to disturb movements of the A domain, whereas p.Glu982Lys may interfere with the function of transmembrane helix TM10 of SERCA1. Protein analyses of affected muscles showed the absence of SERCA1. These results demonstrate that p.Ile235Asn and p.Glu982Lys mutations are disease associated and that the siblings carrying these mutations suffer from Brody myopathy.

A severe reduction in SERCA1 expression and SERCA1 activity has been reported for many Brody patients carrying a number of SERCA1 mutations (Brody 1969; Karpati et al. 1986; Odermatt et al. 2000; Vattemi et al. 2010; Guglielmi et al. 2013). The reduction in SERCA1 expression in Brody patients ranges from 50% to complete loss of the protein. Remarkably, patients, including ours, tolerate this near absence of SERCA1 and they are still able to relax their muscle, albeit at very slow rate. Thus, Brody disease has a mild presentation when muscular activity is paced at tolerable levels, but becomes acute with rapid, repetitive exercise. This suggests the presence of a compensatory Ca^2+^ removal by other functionally similar proteins, including plasma membrane Ca^2+^ ATPases, SERCA2, and SERCA3 (MacLennan et al. 1998; MacLennan 2000). Among these, SERCA2 is likely to play the most significant role in skeletal muscle Ca^2+^ transport. Indeed, SERCA2 expression was increased by approximately twofold in our patient. This upregulation was independently confirmed by confocal microscopy. Immunostaining of affected muscles revealed 2–4 times higher than normal levels SERCA2 staining in both slow and fast myofibers, respectively. The elevated SERCA2 expression in fast fibers lacking SERCA1 clearly demonstrate a compensatory mechanism that partially restores diminished Ca^2+^ transport in Brody patients that are heavily muscled. This might be explained by marked hypertrophy of slow-twitch muscles due to the effort involved in trying to force relaxation on fast-twitch fibers, with little capacity to pump Ca^2+^, by compensatory activation of SERCA2.

Since our affected siblings shared disease-associated mutations in *RYR1* and *ATP2A1*, and the index case was diagnosed as MHS after postoperative rigidity, we considered the possibility that this family is affected with two different coexisting diseases, MH and Brody myopathy. It is well established that pathogenic dominant *RYR1* mutations, when exposed to triggering conditions (anesthetics in humans; stress in swine), induce spontaneous, oscillating Ca^2+^ release that rapidly elevates myoplasmic Ca^2+^ levels, leading to a fulminant MH reaction (Rosenberg et al. 1997; Maclennan and Zvaritch 2011). Such results have been demonstrated with the p.Lys1393Arg mutation expressed in human B cells (Vukcevic et al. 2010), but the larger body of evidence for a role for this mutation in MH in humans is not strongly supportive. This mutation occurs in the general and MHN control populations at a frequency of 1–3% which is well above the estimated frequency of MHS-associated mutations in *RYR1* (Rosenberg et al. 1997; Brandom et al. 2013). There is no clear evidence of segregation of this mutation with MHS phenotype in affected families (Broman et al. 2009; Dlamini et al. 2013; Løseth et al. 2013). The mutation is also predicted to have a negligible impact on protein function by mutation prediction analyses. All of these data, contrary to published reports, indicate that the p.Lys1393Arg is not a pathogenic disease causing mutation; it should be classified as a benign polymorphism. Recent analyses of 850 exomes indicate that a number of *RYR1* polymorphisms have been misclassified as pathogenic mutations in early MH genetic studies (Gonsalves et al. 2013).

We have observed a limited proteolysis of RYR1 that appears to be specific for the RYR1 protein in our Brody family and may result from unrecognized consequences of mutant SERCA1. Depletion of SR (and endoplasmic reticulum [ER]) Ca^2+^ due to mutant SERCA1 could lead to an ER stress response leading to protein aggregation and degradation similar to that evoked by mutations in SERCA2 (Ahn et al. 2003; Wang et al. 2011). However, ER stress leads to progressive disease. The mild presentation of myopathy and a lack of progression of Brody disease would not be expected if an ER stress response were triggered, leading to apoptosis, as is possibly the case in progressive congenital myopathies arising from recessive mutations in *RYR1* (Zvaritch et al. 2009; Maclennan and Zvaritch 2011). Proteolysis of RYR1 likely occurred after the harvest of the tissue. In keeping with this view, there was no obvious impairment of excitation–contraction coupling in studied patients, indicating that protein was fully functional throughout their lives.

It has been reported that some of the Brody patients developed MH symptoms or have been diagnosed as MHS by contracture testing (Karpati et al. 1986; Odermatt et al. 1997; Odermatt et al. 2000; Guglielmi et al. 2013). However, MH symptoms in our Brody patient developed only postoperatively, which is not a common feature of a typical MH episode. While postoperative complications have been reported in some MH patients (Burkman et al. 2007; Larach et al. 2008), MH episode usually develops within the first few hours of exposure to inhalation anesthetics with signs of hypercarbia, tachycardia, and rapid increase in temperature (Burkman et al. 2007; Visoiu et al. 2014). Outside of the operating room, MH is diagnosed by muscle contracture tests (Larach 1989; Ording et al. 1997) that are highly sensitive to abnormal myoplasmic Ca^2+^ regulation. However, the sensitivity of contracture tests is compromised by its low specificity of 78% (Allen et al. 1998). MHS diagnosis in Brody myopathy patients might be due to the 22% false-positive rate of the CHCT bioassay. While there is an obvious difference in clinical presentation, molecular genetics, and disease mechanisms between MH and Brody myopathy, a feature common to both conditions is an elevated myoplasmic Ca^2+^ concentration. We suggest that the cause of the elevated myoplasmic Ca^2+^ that produced abnormal results on CHCT leading the diagnosis of MHS in our index patient was the mutations in SERCA1, not the mutation in RYR1.

In conclusion, we identified a family with Brody myopathy associated with new compound heterozygous mutations in SERCA1. Affected muscle showed near absence of SERCA1 and significant increase in SERCA2 protein level, demonstrating a mechanism that partially restores diminished Ca^2+^ transport. The index case of the family was initially referred for genetic analysis of MH due to postoperative skeletal muscle rigidity; subsequent CHCT led to diagnosis of MHS. We conclude that positive MH contracture responses in vitro or postoperative rigidity with creatine kinase elevation in our Brody patients are due to prolonged high levels of Ca^2+^ as a consequence of compound heterozygous mutations in the *ATP2A1* gene that lead to the lack of SERCA1 protein and SERCA1 Ca^2+^ pumping activity. Our finding of a compensatory SERCA2 upregulation in both slow and fast myofibers lacking SERCA1 protein is novel for Brody disease pathology and may explain a relatively mild disease phenotype developing in the absence of the canonically predominant skeletal muscle isoform – SERCA1. Finally, our work highlights the use of a comprehensive approach in elucidating the pathogenic effects of disease-associated mutations, specifically when multiple mutations are found in disease-associated genes.

## Supporting Information

**Figure S1.** Locations of *ATP1A1* mutations associated with Brody myopathy in the domain structure of SERCA1. Mutations identified in this study are underlined. The first and last amino acids of SERCA1 are in italics. **Figure S2.** RYR1 proteolysis is specific to the Brody patient muscle. (A and B) Western blot analysis of RYR1 protein in the Brody patient muscle (BP) and control muscle samples exposed to various experimental conditions that could result in RYR1 proteolysis. (A) A freshly harvested control muscle sample was split into separate myofiber bundles that were frozen in liquid nitrogen immediately after harvesting (snap frozen) or after exposure to 32 mmol/L caffeine (caffeine); 3% halothane (halothane), and after a prolonged incubation for 3 h at RT in oxygenated Ringer solution, containing 2.5 mmol/L Ca^2+^ (Ringer bathed). The full-length RYR1 protein is revealed as a major band in all control muscle samples, regardless of experimental conditions. α-Actinin was used as a loading control. (B) Western blotting analysis of the Brody patient muscle sample (BP) versus a control (Ctrl) sample that has been thawed and left at room temperature for 2 h prior to analysis. The full-length RYR1 protein is readily observed in the control sample suggesting its relatively low susceptibility to proteolysis even under harsh experimental conditions. **Table S1.** The *ATP2A1* polymorphisms identified in a cohort of 50 MHS subjects.
