# Supplementary material for: Exome analysis identifies Brody myopathy in a family diagnosed with malignant hyperthermia susceptibility
Source: Mol Genet Genomic Med. 2014 Jun 6;2(6):472–83. doi: 10.1002/mgg3.91 (PMC4303217; doi:10.1002/mgg3.91)
Supplement: Supplementary file 1 — Figure S1. Locations of ATP1A1 mutations associated with Brody myopathy in the domain structure of SERCA1. Mutations identified in this study are underlined. The first and last amino acids of SERCA1 are in italics. Figure S2. RYR1 proteolysis is specific to the Brody patient muscle. (A and B) Western blot analysis of RYR1 protein in the Brody patient muscle (BP) and control muscle samples exposed to various experimental conditions that could result in RYR1 proteolysis. (A) A freshly harvested control muscle sample was split into separate myofiber bundles that were frozen in liquid nitrogen immediately after harvesting (snap frozen) or after exposure to 32 mmol/L caffeine (caffeine); 3% halothane (halothane), and after a prolonged incubation for 3 h at RT in oxygenated Ringer solution, containing 2.5 mmol/L Ca2+ (Ringer bathed). The full-length RYR1 protein is revealed as a major band in all control muscle samples, regardless of experimental conditions. α-Actinin was used as a loading control. (B) Western blotting analysis of the Brody patient muscle sample (BP) versus a control (Ctrl) sample that has been thawed and left at room temperature for 2 h prior to analysis. The full-length RYR1 protein is readily observed in the control sample suggesting its relatively low susceptibility to proteolysis even under harsh experimental conditions. Table S1. The ATP2A1 polymorphisms identified in a cohort of 50 MHS subjects. [file mgg30002-0472-sd1.docx]

**Exome sequencing identifies Brody myopathy in a family diagnosed with Malignant Hyperthermia Susceptibility**

Sambuughin, N., Zvaritch, E., Kraeva, N., Sizova, O., Sivak, E., Dickson, K., Weglinski, M., Capacchione, J., Muldoon, S., Riazi, S.,

Hamilton, S., Brandom, B., MacLennan, D.H.

**Supplemental material**

Table 1S. The *ATP2A1* polymorphisms identified in a cohort of 50 MHS subjects

| Variant location | Nucleotide change | cDNA change | Protein change | SNP-ID |
| --- | --- | --- | --- | --- |
| 5’ UTR* | C>T | c.-22C>T | N/A | rs75273069 |
| Intron 1 | C>A | c.118+21C>A | N/A | rs62037371 |
| Intron 4 | CT_del | c.325-47_325-46 | N/A | rs5816477 |
| Exon 8 | C>G | c.663C>G | p.Gly221= | rs113803159 |
| Exon 8 | T>C | c.678T>C | p.Thr226= | rs6565259 |
| Intron 17 | G>T | c.2524+3G>T | N/A | rs2071341 |
| Intron 18 | G>A | c.2610+40G>A | N/A | rs41292394 |
| 3’ UTR* | G>A | c.*1G>A | N/A | rs10499 |

*-Untranslated region

**
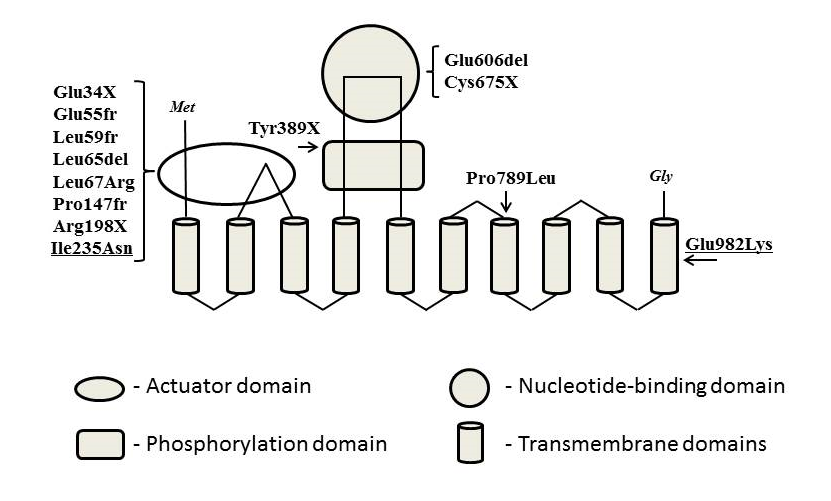
**

**Figure S1.** Locations of *ATP1A1* mutations associated with Brody myopathy in the domain structure of SERCA1.

Mutations identified in this study are underlined. The first and last amino acids of SERCA1 are in italics.

**
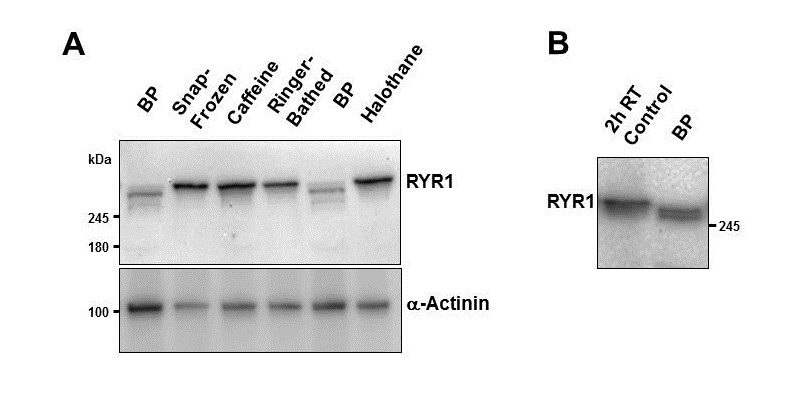
**

**Figure S2.** RYR1 proteolysis is specific to the Brody patient muscle. (**A** and **B**) Western blot analysis of RYR1 protein in the Brody patient muscle (**BP**) and control muscle samples exposed to various experimental conditions that could result in RYR1 proteolysis. **A)** A freshly harvested control muscle sample was split into separate myofiber bundles that were frozen in liquid nitrogen immediately after harvesting (**Snap**-**Frozen**) or after exposure to 32 mM caffeine (**Caffeine**); 3% halothane (**Halothane**), and after a prolonged incubation for 3h at RT in oxygenated Ringer solution, containing 2.5 mM Ca^2+^ (**Ringer-Bathed**). The full-length RYR1 protein is revealed as a major band in all control muscle samples, regardless of experimental conditions. α-Actinin was used as a loading control. **B)** Western blotting analysis of the Brody patient muscle sample (**BP**) *vs* a control (**Ctrl**) sample that has been thawed and left at room temperature for 2 h prior to analysis. The full-length RYR1 protein is readily observed in the control sample suggesting its relatively low susceptibility to proteolysis even under harsh experimental conditions.
